# A gB/CD3 bispecific BiTE antibody construct for targeting Human Cytomegalovirus-infected cells

**DOI:** 10.1038/s41598-018-36055-2

**Published:** 2018-11-28

**Authors:** Charlotte U. Brey, Julia Proff, Natascha Teufert, Benjamin Salzer, Johannes Brozy, Markus Münz, Jochen Pendzialek, Armin Ensser, Wolfgang Holter, Manfred Lehner

**Affiliations:** 1 Children’s Cancer Research Institute, Vienna, Austria; 2 Institute for Clinical and Molecular Virology, Universitätsklinikum Erlangen, Friedrich-Alexander-Universität Erlangen-Nürnberg, Erlangen, Germany; 3 AMGEN Research (Munich) GmbH, Munich, Germany; 4 St. Anna Kinderspital, Department of Pediatrics, Medical University of Vienna, Vienna, Austria

## Abstract

Bispecific T cell engager (BiTE) antibody constructs are successfully used as cancer therapeutics. We hypothesized that this treatment strategy could also be applicable for therapy of human cytomegalovirus (HCMV) infection, since HCMV-encoded proteins are abundantly expressed on the surface of infected cells. Here we show that a BiTE antibody construct directed against HCMV glycoprotein B (gB) and CD3 efficiently triggers T cells to secrete IFN-γ and TNF upon co-culture with fibroblasts infected with HCMV strain AD169, Towne or Toledo. Titration of gB expression levels in non-infected cells confirmed that already low levels of gB are sufficient for efficient triggering of T cells in presence of the BiTE antibody construct. Comparison of redirecting T cells with the bispecific antibody versus a chimeric antigen receptor (CAR) based on the same scFv showed a similar sensitivity for gB expression. Although lysis of infected target cells was absent, the BiTE antibody construct inhibited HCMV replication by triggering cytokine production. Notably, even strongly diluted supernatants of the activated T cells efficiently blocked the replication of HCMV in infected primary fibroblasts. In summary, our data prove the functionality of the first BiTE antibody construct targeting an HCMV-encoded glycoprotein for inhibiting HCMV replication in infected cells.

## Introduction

The reactivation of human cytomegalovirus (HCMV) remains a major cause of morbidity and mortality after allogeneic hematopoietic stem cell transplantation (HSCT)^1,2^. This is a particular problem in the high-risk constellation of an HCMV seronegative donor and an HCMV seropositive recipient, where about 80% of the transplanted patients develop viremia^3–5^. Typically, early reactivation of HCMV is associated with an increased risk of developing graft versus host disease (GVHD) and additional bacterial and fungal infections^6,7^. Thus, pre-emptive therapy with ganciclovir or its oral prodrug valganciclovir as first-line treatment is started as soon as virus load is detected in the blood in order to prevent progression of the asymptomatic infection. However, this treatment has significant bone marrow toxicity and drug resistance may develop with these drugs or also with foscarnet or cidofovir used in second-line treatment^7,8^. Drug resistance, again, requires prolonged antiviral treatment and is associated with poorer outcome^9,10^. Currently, several new antiviral drugs are investigated in clinical trials, however, also the new drugs are likely to become associated with the development of resistance and toxicities limiting their clinical applicability^7,10,11^.

One of the clinically most advanced immunotherapy approaches in cancer therapy uses Bispecific T cell engagers (BiTE), which are bispecific antibody constructs consisting of two single-chain variable fragments (scFv) connected by a short linker. One scFv is antigen-specific, whereas the other one targets CD3 on T cells. Thereby, BiTE antibody constructs redirect T cells to the target cell, engaging the T cell effector functions and eliciting cell lysis^12,13^. The first BiTE antibody construct that was approved by the FDA in December 2014 was blinatumomab (Blincyto), a BiTE antibody construct directed against CD19, which is expressed on the surface of B cells. Blinatumomab is successfully used for the treatment of acute lymphoblastic leukaemia (ALL) in paediatric and adult patients^14^. Different BiTE antibody constructs are in preclinical and clinical investigation, targeting antigens in solid tumours (CEA, PSMA) as well as hematopoietic malignancies (CD33)^12,13^. Here, we test a BiTE antibody construct as a new approach for HCMV therapy.

HCMV infection is a potential target for a BiTE approach since several glycoproteins encoded by HCMV, among them gB as the best studied, are abundantly expressed on the surface of infected cells as intact proteins. In addition, gB is the most highly conserved glycoprotein with a reported sequence homology between strains of 88.16–99.89% making it a promising antigen to target^15^. In a previous work we have therefore constructed a gB specific CAR^16^, which is based on a scFv of the monoclonal antibody clone 27–287 and targets a highly conserved region within the antigenic domain 1 (AD-1) of the gB ectodomain^15,17,18^. T cells expressing the CAR were specifically activated in response to HCMV-infected cells, thus demonstrating the potential of targeting gB as an antigen^16,19^.

The clinical implementation of a gB targeted T cell therapy would certainly be facilitated by a strategy based on a bispecific antibody approach due to safety aspects and simplified production compared to CAR-T cells. Such a strategy was recently reported by Meng *et al*.^20^. We pursued an approach based on the well-established BiTE technology and generated a BiTE antibody construct directed against HCMV gB. In our study, we tested this construct with primary fibroblasts infected with different HCMV strains, compared it with CAR-T cells, and demonstrated that it can limit the spread of HCMV independently of cytolysis.

## Results

### Titration of the gB specific BiTE antibody construct

The first aim was to determine a suitable concentration of our gB-BiTE antibody construct for concomitantly enabling maximum activation of T cells in the presence of the targeted antigen and a minimum of nonspecific activation in the absence of the latter. For this purpose, we tested concentrations of the antibody construct ranging from 0.1 ng/ml up to 1000 ng/ml, which have been reported in the literature and are effective with other BiTE antibody constructs^21–23^.

**Figure 1:**
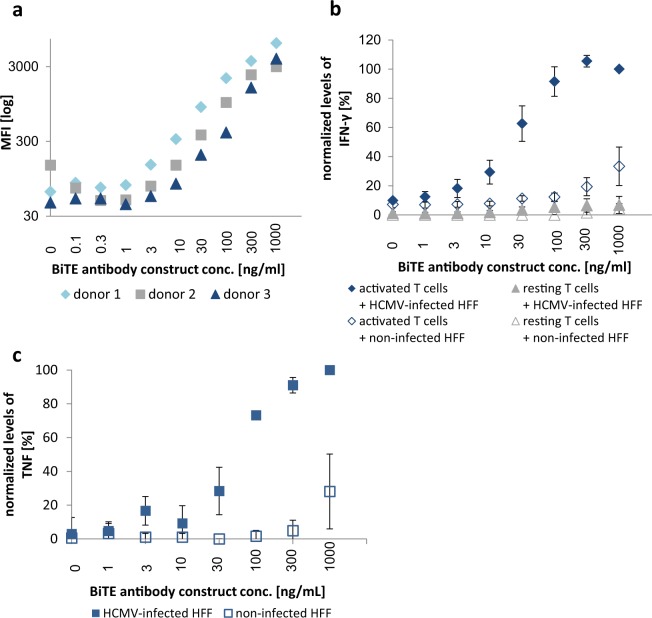
Titration of the gB-BiTE antibody construct. (**a**) Flow cytometric analysis of the amounts of BiTE antibody construct bound to T cells, which were previously activated and expanded by anti-CD3/CD28-antibody coated beads. (**b**) Normalized levels of IFN-γ secreted by activated or resting T cells, respectively, upon co-culture with non-infected or HCMV-infected HFF (AD169, MOI 5, day 4 p.i.) and an increasing amount of the BiTE antibody construct (mean ± standard deviation; n = 3). (**c**) Normalized levels of TNF secreted by CD3/CD28-activated T cells upon co-culture with non-infected or HCMV-infected HFF (AD169, MOI 5, day 4 p.i.) and an increasing concentration of the BiTE antibody construct (mean ± standard deviation; n = 3). IFN-γ and TNF levels were normalized for each donor to the value obtained with activated T cells co-cultured with HCMV-infected HFF and 1000 ng/ml BiTE antibody construct.

In a first step we determined the saturating concentration of the gB specific BiTE antibody construct required for binding to CD3/CD28-activated T cells. For this purpose, the antibody construct was added in different concentrations and the amounts of the bound construct were quantified via its integrated Histidine-Tag (His-Tag) using flow cytometry (Fig. 1a). Binding of the antibody construct in our experimental setting was detectable starting from 3 ng/ml and approached saturation at the highest concentration of 1000 ng/ml. Next, we performed co-culture experiments using either resting or previously CD3/CD28-activated T cells as effector cells and HCMV-infected or non-infected human foreskin fibroblasts (HFF) as targets. The expression of gB on the surface of HCMV-infected HFF is shown in Supplemental Fig. 1a, and the phenotype of CD3/CD28-activated T cells is shown in Supplemental Fig. 1b,c. The phenotype of the expanded T cells was similar for all three donors with a roughly comparable ratio of CD4^pos^/CD8^pos^ T cells (0.9–1.93; representative dotplot shown in Supplemental Fig. 1c). Viable cells consisted of 96.5–99.6% CD3^pos^ T cells and did not contain CD3^neg^/CD56^pos^ NK cells (data not shown). The activation of these T cells by the gB specific BiTE antibody construct was quantified by measuring the secreted cytokines IFN-γ and TNF, as these cytokines are known to synergistically mediate the inhibition of HCMV replication^24–27^. This previously reported synergistic effect was confirmed by us originally by adding IFN-γ and TNF to infected HFF (Supplemental Fig. 2) and in our recent study with gB specific CAR-T cells also by blocking IFN-γ and TNF in T cell supernatants^28^.

The gB-BiTE antibody construct triggered T cells in co-cultures with infected HFF starting from around 10 ng/ml and reached a maximum at about 300 ng/ml (Fig. 1b). At this concentration, the highest levels of secreted IFN-γ were obtained with CD3/CD28-activated T cells of all three donors (ranging from 4.6 to 35.9 ng/ml, shown in Fig. 1b as normalized values). The same correlation was observed for TNF, although the amounts overall were lower (maximum levels ranging from 104 to 230 pg/ml, shown in Fig. 1c as normalized values) and the levels secreted by resting T cells were below the detection limit. Target antigen independent triggering of cytokine secretion from the T cells in co-cultures with non-infected HFF was observed starting from about 100 to 300 ng/ml of the antibody. Resting T cells released much lower levels of IFN-γ in response to HCMV-infected HFF than activated T cells. Hence, the values obtained with resting T cells (maximum levels ranging from 0.12 to 2.4 ng/ml) are close to the base line in Fig. 1b due to normalization to the maximum values obtained with the CD3/CD28-activated T cells. The concentrations of IFN-γ secreted by CD3/CD28-activated and resting T cells are shown with one donor for illustration (Supplemental Fig. 3a,b).

After this first set of experiments, we chose 300 ng/ml of the gB-BiTE antibody construct as standard working concentration, since this concentration seemed to be saturating and higher levels were associated with increased antigen-independent T cell activation. Furthermore, all following experiments were performed with CD3/CD28-expanded T cells.

### Functional comparison of the BiTE antibody construct with a gB specific CAR

**Figure 2:**
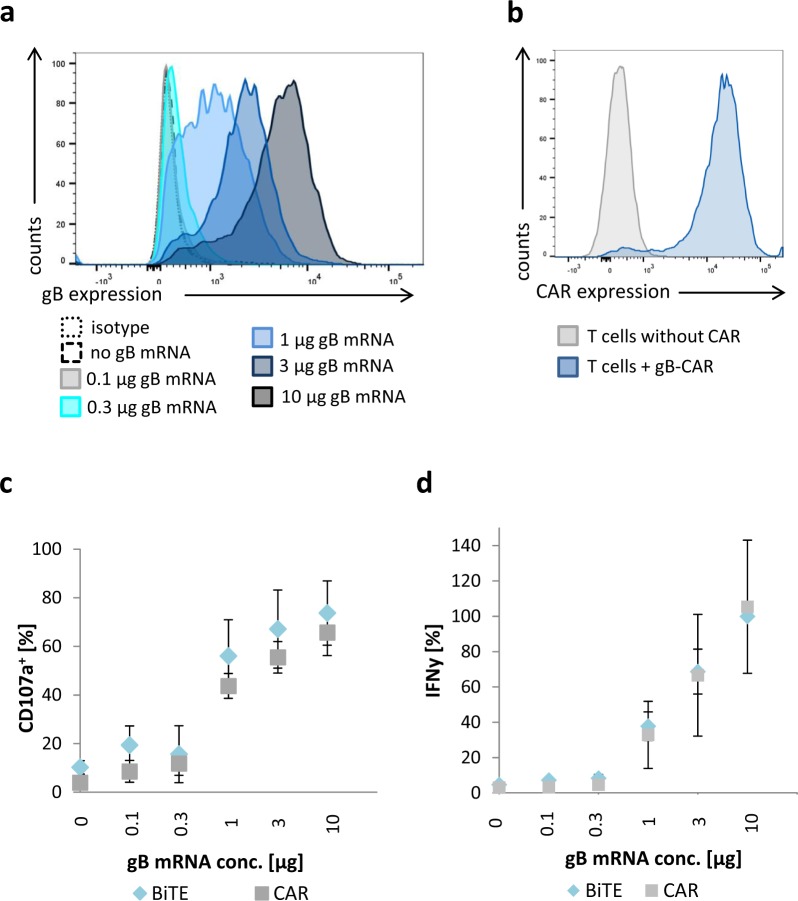
Comparison of the efficiency of redirecting T cells to gB by either the BiTE antibody construct or a CAR. Activated T cells were redirected by either the gB-BiTE antibody construct or a gB-CAR and co-cultured with 293T cells, which were electroporated with increasing amounts of chimeric EpCAM-gB mRNA, as indicated. (**a**) Expression levels of EpCAM-gB in the target cells 293T 18 hours after electroporation with different amounts of EpCAM-gB mRNA. (**b**) Expression of the gB-CAR in CD3/CD28-activated primary T cells of one representative experiment. (**c**) Proportion of degranulating T cells as determined by flow cytometric analysis of cell surface expression of CD107a. (**d**) Normalized levels of secreted IFN-γ (values obtained with gB-BiTE antibody construct at 10 µg EpCAM-gB mRNA were set to 100%). (**c**,**d**) display the mean values ± standard deviation of experiments with three donors.

After determining the optimal concentration of the gB-BiTE antibody construct, we went on with a functional comparison of the protein side by side with a previously characterized gB specific CAR based on the same scFv^16,19^. Therefore, we analysed the activation of T cells in response to target cells expressing a fusion construct of gB from low up to high levels. Hereto, 293T cells were electroporated with different amounts of mRNA encoding the gB fusion construct, which resulted in detectable expression starting from 1 μg of mRNA (Fig. 2a). For expression of the gB specific CAR, the T cells were electroporated with the respective mRNA resulting in >95% of CAR^pos^ T cells (Fig. 2b). Both the gB specific CAR and the gB specific BiTE antibody construct were able to trigger degranulation in a comparable fraction of CD3/CD28-activated T cells when co-cultured with 293T cells expressing different levels of recombinant gB (Fig. 2c). For example, 293T cells electroporated with 1 μg gB mRNA elicited degranulation in 36.6–72.8% of the T cells redirected by the BiTE antibody construct and in 37.0–49.4% of the T cells redirected by the CAR. Comparable performance of the gB-BiTE antibody construct was also observed when analysing the IFN-γ levels (Fig. 2d). T cells redirected by the bispecific antibody secreted 31.1–49.3% of the maximum IFN-γ levels (corresponds to 0.66–1.8 ng/ml IFN-γ) and T cells redirected by the CAR secreted 17.2–59.7% of the maximum IFN-γ levels (corresponds to 0.36–2.1 ng/ml IFN-γ) when using target cells electroporated for example with 1 µg of gB mRNA. Degranulation of T cells and IFN-γ secretion increased only starting from 1 μg mRNA, which correlated with detectable expression of recombinant gB (Fig. 2a). Interestingly, despite an about 10-fold difference in gB expression levels obtained with 1 vs. 10 μg mRNA, the degranulation and IFN-γ levels were only moderately increased by comparison. For example, with 1 μg of gB mRNA, the gB-BiTE antibody construct could trigger already 38% of the maximum IFN-γ levels obtained with 10 µg gB-mRNA.

Taken together, these experiments clearly show that the tested gB-BiTE antibody construct is effective, comparable to its related CAR construct in redirecting T cells to gB. The data also show that strong T cell activation by the gB-BiTE antibody construct occurs when the lowest detectable levels of gB are expressed on the target cell surface.

### Recognition of target cells infected with different HCMV strains

**Figure 3:**
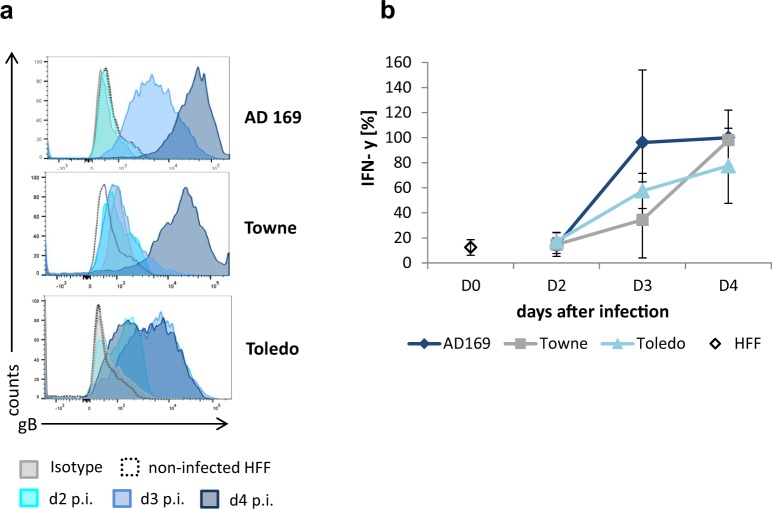
Recognition of fibroblasts at different time points after infection with different HCMV strains. (**a**) Expression levels of gB in HFF within 4 days after infection with the HCMV strains AD169, Towne and Toledo (MOI 1). Shown is one representative experiment. (**b**) Normalized levels of IFN-γ secreted by CD3/28 activated T cells, which were redirected by the gB-BiTE antibody construct and co-cultured with HFF at different time points after infection with the indicated HCMV strains (mean ± standard deviation; n = 3).

As the parental antibody 27–287 is directed against a highly conserved epitope within the AD-1 domain of gB we further explored whether our gB-BiTE antibody construct could therefore mediate recognition of target cells infected with different HCMV strains. Hereto, we tested the three commonly used laboratory strains AD169, Towne and Toledo. The expression of gB was analysed on days 2, 3 and 4 after infection (MOI 1) by using the parental antibody 27–287. This confirmed the recognition of the epitope and, interestingly, revealed different kinetics in the occurrence of this protein on the surface of the infected cells (Fig. 3a). Surface expression of gB was moderate or absent on day 2 and thereafter increased with different kinetics and amplitude. With the strain Toledo, gB expression reached its peak already on the third day, but was characterized by a reduced peak level compared to Towne and AD169. With the latter two strains, gB expression increased until day 4 and reached high levels. Of all three strains, Towne was characterized by the most delayed increase in gB expression. These differences in the kinetics and the amplitude of gB expression were roughly reflected in the capability of the target cells to activate the T cells redirected by the BiTE antibody construct (Fig. 3b). In the shown experiments, the T cells were co-cultured for 4 hours with the HFF at different time points after HCMV infection at an effector:target (E:T) ratio of 2:1 in the presence of 300 ng/ml gB-BiTE antibody construct. Analysis of the supernatants revealed high amounts of secreted IFN-γ (7–22 ng/ml; i.e., the maximum IFN-γ levels used for normalization in Fig. 3b) in the co-cultures with HFF harvested 4 days after infection and only moderate differences among the three HCMV strains. Overall, the expression of gB correlates with the levels of secreted cytokines and confirms that the gB-BiTE antibody construct efficiently recognizes gB from different HCMV strains starting from day 3 after infection.

### Cytokine mediated inhibition of HCMV replication by the gB-BiTE antibody construct

**Figure 4:**
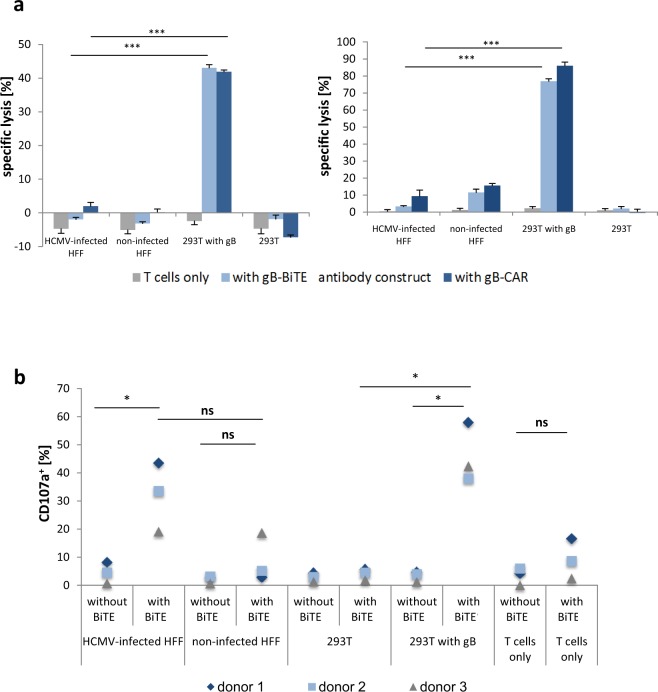
Triggering of cytotoxic effector functions by the gB-BiTE antibody construct. (**a**) Activated T cells were co-cultured with different target cells at an E:T ratio of 25:1 for 4 hours. The target cells 293T were either electroporated with mRNA encoding EpCAM-gB or were mock electroporated. HFF were used for experiments on day 4 after infection (AD169, MOI 5), non-infected cells were used as a control. Data of two representative donors are shown. (**b**) Proportion of degranulating T cells (characterized by CD107a surface expression) upon co-culture with different target cells as indicated (n = 3).

In our previous study with gB specific CAR-T cells we could demonstrate that HCMV-infected cells are strongly resistant to T cell mediated lysis on day 3 and 4 after infection, i.e., when gB occurs on the cell surface. This effect is independent of the well-known down-regulation of MHC I molecules, but is at least partially caused by HCMV-encoded anti-apoptotic proteins like UL36 and UL37x1^19^. Indeed, we could observe this type of resistance to T cell mediated cytotoxicity also in our experiments with T cells redirected by the gB-BiTE antibody construct. As previously observed with the CAR-T cells, HCMV-infected cells (AD169, MOI 5, day 4 p.i.) could not be lysed by T cells from two different donors, whereas non-infected target cells expressing gB were very efficiently lysed (Fig. 4a). Our experiments with the BiTE antibody construct also confirmed that resistance to T cell lysis occurs despite effective degranulation of the T cells (Fig. 4b).

**Figure 5:**
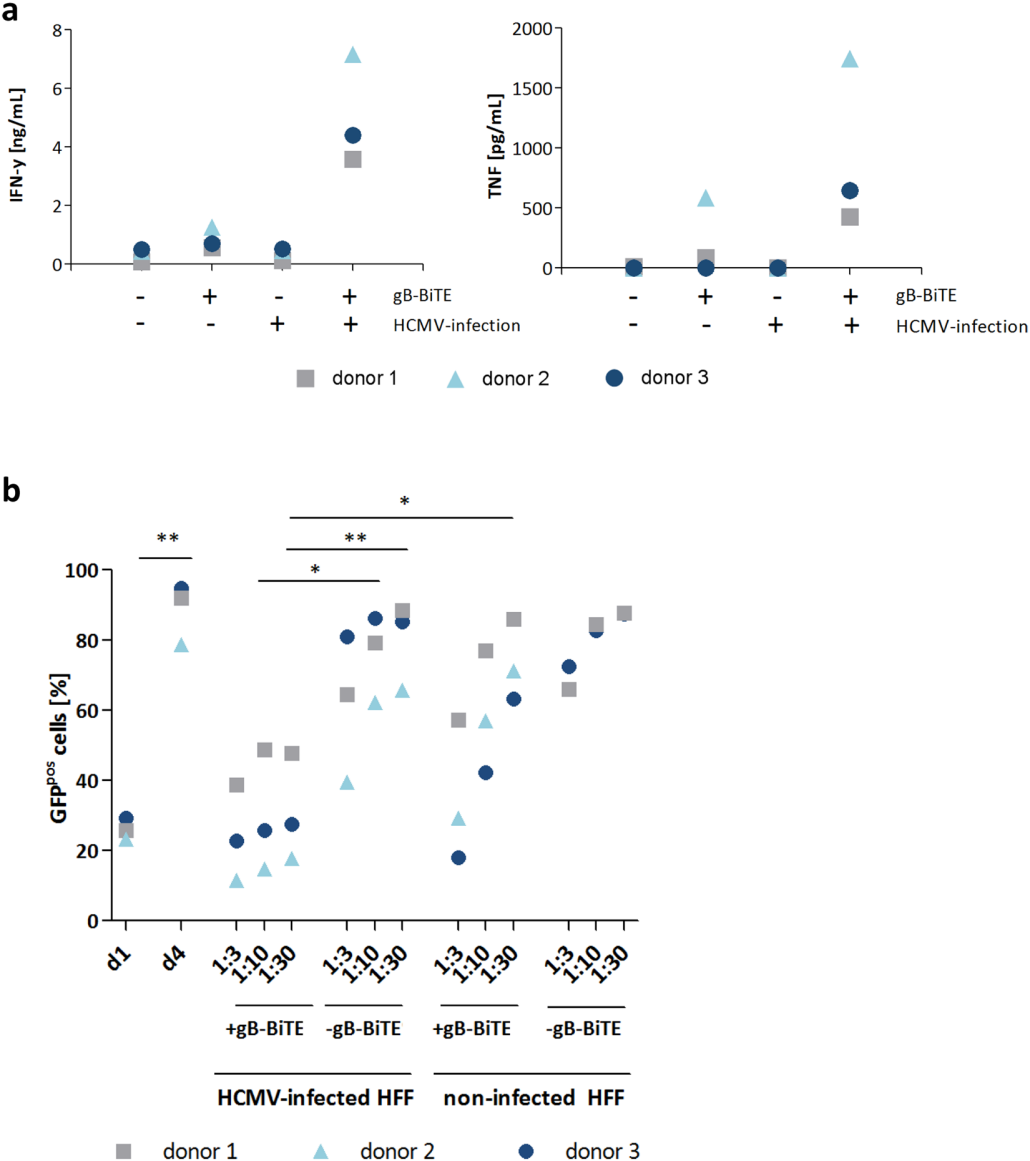
Inhibition of HCMV infection in HFF by supernatants of T cells redirected by the gB-BiTE antibody construct. (**a**) Levels of IFN-γ and TNF secreted by activated T cells with or without BiTE antibody construct after co-culture with HFF, non-infected or infected with HCMV (AD169, MOI 5, day 4 p.i.), as indicated (n = 3). For the experiments shown in **(b)**, serial dilutions (1:3, 1:10, 1:30) of the supernatants of the co-cultures analysed in (**a**) were added to HFF throughout and after the infection with HCMV (AD169, MOI 0.25). The proportion of infected HFF was then analysed 4 days later by flow cytometric detection of GFP expression (n = 3; n = 2 with the supernatants from non-infected HFF without BiTE antibody construct). Additionally, control cultures without added supernatants were analysed for the proportion of infected cells one day after infection. The increase of GFP^pos^ cells until day 4 beyond these reference levels on day one indicates the fraction of cells, which were infected by the newly generated HCMV particles.

In our study with the CAR-T cells, we have further observed that viral replication in infected target cells can still be efficiently inhibited by the synergistic action of IFN-γ and TNF, which both were strongly secreted upon activation via the gB-CAR^28^. In this recent study we also showed with neutralizing antibodies that the simultaneous blockade of IFN-γ and TNF strongly reduces the inhibitory function of the CAR-T cell supernatants. This inhibitory effect of IFN-γ and TNF on HCMV is well known and was also confirmed by us earlier by the addition of the two cytokines to infected HFF (Supplemental Fig. 2)^24–27^. Therefore, we have here investigated the efficiency of the gB specific BiTE antibody construct in cytokine mediated inhibition of HCMV replication. For this purpose, supernatants from 4 different conditions (CD3/CD28-expanded T cells +/− gB-BiTE antibody construct and co-cultured with non-infected or HCMV-infected HFF) were generated for subsequent analysis of their capacity for inhibiting HCMV replication in freshly infected HFF. Figure 5a shows the levels of IFN-γ and TNF detected in these supernatants. Addition of the gB-BiTE antibody construct triggered low unspecific IFN-γ secretion (0.56–1.27 ng/ml) in co-cultures with non-infected HFF and strong specific secretion (3.58–7.16 ng/ml) in co-cultures with HCMV-infected HFF. These cytokine-containing supernatants were added in different dilutions (ranging from 1:3 to 1:30) to HFF throughout and after infection (AD169, MOI 0.25, infectious supernatant removed after 4 hours by two times washing). This yielded an infection rate of 23.4–29.4% (Fig. 5b), as quantified on day 1 p.i. by the fraction of HFF expressing GFP, which was encoded by the recombinant AD169 and driven by an immediate early HCMV promoter. The increase of the fraction of GFP^pos^ HFF from day 1 until day 4 (78.7–94.7%) is caused by infection of the remaining non-infected HFF by newly generated HCMV particles. In accordance with a previous report^29^, the addition of cytokine containing supernatant could not inhibit HCMV replication in already-infected cells. However, the supernatant almost completely blocked the replication of newly released HCMV in the initially non-infected cells, as can be seen in the proportion of infected cells that is similar to the level of infection observed on day 1 (Fig. 5b). This was also observed with the highest dilution of 1:30 with all three donors (17.9–47.7% GFP^pos^ cells on day 4 p.i.). This inhibition was statistically significant when compared to the values obtained with supernatants from co-cultures of infected HFF and T cells without BiTE antibody construct and also with supernatants of co-cultures containing the BiTE antibody construct but with non-infected HFF. The latter also proves that the observed blockade of HCMV replication was mediated by the cytokines and not directly by the BiTE antibody construct.

In summary, these data indicate that directing T cells to gB using the BiTE antibody construct enables efficient cytokine mediated inhibition of HCMV replication, which is in agreement to our data previously obtained with gB-CAR expressing T cells.

## Discussion

A range of BiTE antibody constructs and other bispecific antibody formats have entered the clinic. Of those, blinatumomab has demonstrated efficacy in hematologic malignancies^12,13,30^. Similarly, HLA independent targeting of virus-infected cells by bispecific antibodies or CARs has been pursued and reported for HIV, HBV, HCV, and recently also HCMV^20,31–34^. We characterized a gB specific antibody construct based on the BiTE technology and showed that HCMV infection, despite resistance mechanisms to cytotoxic effector functions, could potentially be a target for a bispecific antibody approach.

Fibroblasts infected with the HCMV strain AD169 express high levels of gB already 3 days after infection and T cells in co-culture start secreting IFN-γ and TNF upon addition of a gB specific BiTE antibody construct. We could show this with resting T cells and also with T cells expanded by CD3/CD28-beads, whereby the latter T cells released more than a log-scale higher amounts of IFN-γ (Fig. 1b and Supplemental Fig. 3). Generally, the amounts of secreted IFN-γ were markedly higher than that of TNF (Fig. 5a), which is in agreement with previous observations with naturally occurring HCMV reactive CD4^pos^ T cell clones^24^. At the saturating concentration of 300 ng/ml of the BiTE antibody construct IFN-γ was released at similarly high levels as from gB specific CAR-T cells (Fig. 2d). This is notable given the fact that the CAR via its integrated CD28 domain triggers co-stimulation, whereas the antibody construct does not. Furthermore, titration of the gB expression levels in 293T target cells showed that the BiTE antibody construct has comparable sensitivity for gB expression as the related gB specific CAR with regard to triggering degranulation and cytokine production in the T cells. Both the antibody construct and the CAR triggered degranulation and cytokine release when the lowest gB levels detectable by the parent antibody (clone 27-287) were expressed on the surface of transfected cells. Above a certain threshold of gB expression, the further increase in degranulation and cytokine release was only moderate (Fig. 2a,c,d).

The cytokines IFN-γ and TNF are known to synergize in triggering an antiviral state in different cell types, which blocks replication of HCMV and also of other viruses^24–27^. We have previously shown that IFN-γ and TNF released from gB specific CAR-T cells can strongly inhibit HCMV replication^28^. In the present study we were able to reinforce this observation with the BiTE antibody construct. In our experiments, 30-fold dilutions of co-culture supernatants of T cells directed by the BiTE antibody against HCMV-infected HFF almost completely suppressed HCMV replication, while the same dilutions of co-culture supernatants of T cells directed by the antibody against non-infected fibroblasts showed hardly any inhibition (Fig. 5b). In our previous study with CAR-T cells, we demonstrated by using neutralizing antibodies that the inhibitory effect of T cell supernatants is largely mediated by the two cytokines IFN-γ and TNF^28^. In addition to these two cytokines, a contribution of the higher concentrated BiTE antibody construct present in the less diluted supernatants to inhibition is possible. Although the BiTE antibody construct is directed towards a non-neutralizing epitope, AD1 in gB^17^, such an effect is conceivable since an inhibitory effect was observed *in vivo* even with non-neutralizing antibodies^35,36^. However, the fact that no direct correlate of protection could be found *in vivo* for the AD1 epitope and that the decreased incidence of viremia was correlated in a study with higher antibody levels against AD2 but not with antibody levels against AD1, 4 or 5 makes this possibility rather unlikely^35,37^. The most likely cause of the inhibition observed in T cell supernatants from co-cultures with non-infected HFF is, in fact, the low target-independent activation of T cells by the high concentrations of the BiTE antibody construct used (300 ng/ml). This target-independent T cell activation led to 0.18–0.42 ng/ml IFN-γ in the 1:3 dilutions of the supernatants and was thus still within the effective range (the strongly inhibitory 1:30 dilutions of supernatants from the co-cultures with infected HFF contained 0.12–0.24 ng/ml IFN-γ).

The importance of the IFN-γ and TNF mediated antiviral effect of the BiTE antibody construct is based on the fact that the occurrence of gB at the surface of infected cells coincides with the development of their resistance to T cell cytotoxicity^19^ (Fig. 4a). In our previous work we have shown that this type of resistance is at least partially mediated by direct neutralization of cytotoxic T cell functions by viral anti-apoptotic proteins like UL37x1 and UL36, and as we have already observed with the CAR-T cells we now found that also the BiTE antibody construct could not mediate lysis of HCMV-infected cells. Importantly, however, there is a substantial body of evidence that non-cytolytic effector functions play an important role in limiting viral infections^38–41^. For HCMV the antiviral interferon response is well investigated and the strong antiviral effect of IFN-γ was early demonstrated also *in vivo*^42,43^. Furthermore, gene polymorphism for IFN-γ was correlated with increased susceptibility to HCMV infection in patients after organ transplantation^44,45^. Taken together, these data and our work add to the increasingly recognized view that control of HCMV infection by cellular immunity is also critically dependent on secreted cytokines.

As mentioned earlier the scFv in the BiTE antibody construct was derived from the same antibody clone as the scFv in our previously developed gB specific CAR construct^16^. This antibody clone 27–287 is directed against a non-neutralizing epitope within AD-1 of gB^17^, which is highly conserved among different HCMV strains and clinical isolates^15^, and even in the rhesus CMV^46^. The AD-1 is located on a membrane-distal part of gB and is less glycosylated than other parts of the protein with only two described N-glycosylation sites. The predicted epitope for the anti-AD-1 antibody clone 27-287 is located between amino acids 608 and 625^17^. Within this region, among sixty sequenced clinical and laboratory HCMV strains, there is a single position with two alternative amino acids (Leucine or Phenylalanine at position 611)^15^ which both were also represented by our tested laboratory strains AD169, Towne and Toledo. Notably, the BiTE antibody construct enabled the recognition of cells infected with all three different HCMV strains. These strains are characterized by various genomic alterations^47,48^ and in our hands displayed different kinetics in the occurrence of gB on the cell surface as well as different maximum levels of the protein (Fig. 3a). The activation level of the T cells stimulated by these infected cells in presence of the bispecific antibody thereby approximately correlated with the expression level of gB, respectively (Fig. 3b). Some differences in the released amounts of IFN-γ were observed in co-cultures of T cells and HFF on day 3 after infection, on which gB expression was most variable among the HCMV strains. Four days after infection, however, we observed high gB expression and strong IFN-γ production with all strains.

Together with the high safety profile of the bispecific antibody therapy, our data suggest that the administration of a gB targeted bispecific antibody, such as our BiTE antibody construct or the construct reported by Meng *et al*.^20^, in principle is an attractive option. In particular, the application of such an antibody would be very attractive after HSCT in the high-risk constellation of an HCMV seropositive recipient and an HCMV seronegative donor, when HCMV reactivates after T cells from the donor have already engrafted. In this situation the T cells are naïve and thus not protective, however, they could be directed to infected cells by administration of the bispecific antibody. For further investigation of the *in vivo* efficacy of our bispecific antibody approach, we have initiated a study in which we adapt the system for use in a murine CMV model. In this model, the HCMV gB is ectopically expressed in MCMV under control of the CMV IE1 promoter. Preliminary *in vitro* data show that BiTE retargeted T cells specifically restrict MCMV replication in murine fibroblasts (Supplemental Fig. 4). However, the biology of the murine virus is characterized by a faster replication cycle and differences in the regulation of gB. This model, therefore, cannot provide a definitive proof or disproof of efficacy in HCMV infection. Instead, much more reliable models could be the closely related CMVs of rhesus or cynomolgus monkey, which would also better address safety issues. It could be that T cell activation by the BiTE antibody construct could potentially augment an underlying T cell alloreactivity similarly to what has likely been observed in patients receiving activated NK cells after HSCT^49^. However, the action of BiTE antibody constructs due to short plasma half-life is easier to control than that of a cellular product. Finally, when considering the experience with adoptive transfer of enriched virus-specific T cells after HSCT, although not directly comparable, the danger of triggering GVHD by T cell activation via the antibody at proper dosage might be moderate^50–52^. In this context, it should be noted that the high concentrations of the BiTE antibody construct used in our experiments (300 ng/ml) triggered a weak target antigen-independent T cell activation (Figs 1b,c, 4b and 5a). In the clinical use, such activation could be equivalent to low systemic T cell activation, which could potentially provoke or fuel GVHD. Therefore, the target-independent T cell activation in our experiments is a very important observation to be considered in the preclinical and clinical evaluation of the safety of the approach.

Taken together, our data show that the gB-specific BiTE antibody construct can efficiently redirect T cells to infected cells. Since the construct is direct against a highly conserved epitope of gB it can mediate recognition of cells infected with different HCMV strains. Despite mounting evidence that HCMV infected cells are efficiently protected from T cell-mediated cytotoxicity (*in vitro* and probably *in vivo*), the gB-BiTE antibody construct could, nevertheless, limit HCMV replication via non-cytolytic mechanisms.

## Material and Methods

### Cell culture

The isolation of PBMCs from leukapheresis obtained from voluntary healthy donors was performed by Ficoll density gradient centrifugation. The further isolation of T cells from thawed PBMCs was carried out by negative selection of CD3^pos^ cells using the Dynabeads Untouched Human T cell Kit (Life Technologies) according to the manufacturer’s instructions. Primary HFF were isolated from foreskin of circumcised donors after mechanic disruption by enzymatic digestion with 5 mg/ml Collagenase D, 25 U/ml Dispase, and 0.05% Trypsin/EDTA. HFF were cultured in R-10 medium, consisting of RPMI GlutaMAX (Life Technologies) supplemented with 10% FCS (Sigma-Aldrich), 100 U/ml penicillin and 100 µg/ml streptomycin (both Life Technologies). For the experiments, the HFF were used from passage 10–18. For both, isolation of PBMCs and HFF, written informed consent was obtained from every voluntary donor. All experiments were performed in accordance with the ethical guidelines of the Declaration of Helsinki and approval by the local ethics committees (Friedrich-Alexander-Universität Erlangen-Nürnberg no. 2247, Medizinische Universität Wien no. 514/2011). 293T cells were cultured in DMEM (Life Technologies) supplemented with 10% FCS and 100 U/ml penicillin and 100 µg/ml streptomycin. The hybridoma cell line “gB 27-287” was cultured in R-10 with the same supplements.

### Expansion of T cells

T cells were cultured in R-10 supplemented with 200 U/ml IL-2 (Peprotech) and activated by Human T-Activator CD3/CD28 Dynabeads (Life technologies) with a bead:cell ratio of 1:1 (25 µL of bead suspension containing 1 × 10^6^ beads for 1 × 10^6^ cells in 1 ml seeding volume). T cells were split every second day and were grown between a density of 0.5–1 × 10^6^ cells/ml. Activated T cells were used for experiments between day 21 and 28 after activation. Resting T cells were obtained by isolation of T cells from freshly thawed PBMCs with the Untouched Human T cell Kit (Life Technologies). Isolated resting T cells were cultured overnight in R-10 supplemented with 200 U/ml IL-2.

### Viruses

The GFP encoding recombinant HCMV strain AD169 was a kind gift from M. Marschall (Universitätsklinikum Erlangen, Erlangen, Germany), the GFP encoding recombinant HCMV strains Towne^47^ and Toledo were provided by B. Plachter (Universitätsmedizin, Johannes Gutenberg-Universität Mainz, Mainz, Germany) and B. Torok-Storb (Fred Hutchinson Cancer Research Center, Seattle, USA), respectively. Infectious virus supernatants were generated by infection of semi-confluent HFF layers with the respective HCMV strains (MOI 0.1). The cells were cultured for 10 to 14 days and the supernatants were harvested, centrifuged (2000 rpm, 10 min, 4 °C) and stored at −80 °C until use. The titers of the supernatants were determined by the limiting dilution method according to Reed and Munch^53^. For the experiments, HFF were infected with the respective HCMV strains at an MOI between 0.25 and 5, as indicated, and used between day 2 and day 4 after infection. The appropriate amount of viral supernatant required to reach the desired MOI was added to the cells and after 4 h of incubation at 37 °C, the HFF were washed three times with RPMI GlutaMAX (Life Technologies) to remove remaining viral particles. Afterwards, fresh R-10 medium was added and the cells were further kept at 37 °C.

### Flow cytometric analysis

Cells were counted by flow cytometric analysis using Accucheck counting beads (Life Technologies) and propidium iodide for exclusion of dead cells. Analysis of the T cell phenotype was performed using the following antibodies: CD3 (clone SK7, BD biosciences), CD4 (clone OKT4, eBioscience), CD8 (clone RPA-T8, BD biosciences), CD56 (clone NCAM1.2, BD biosciences). The gB-BiTE antibody construct was detected via the His-tag using an anti-His antibody (clone GG11-8F3.5.1, Miltenyi Biotec). Expression of the gB-CAR in T cells was detected with a biotinylated anti-human IgG mab (clone JDC-10, Southern Biotec) and subsequent staining with PE-conjugated streptavidin (eBioscience). Staining of HCMV-gB was performed with an antibody obtained from supernatants of the hybridoma cell line gB-27-287^17^, which also served for construction of the scFv used in the CAR and the BiTE antibody construct. Binding of this antibody was detected by secondary staining with a PE-conjugated anti-mouse antibody (eBioscience). Non-infected and HCMV-infected HFF were blocked before antibody staining by pre-incubation with 10% human serum from an HCMV-seronegative individual (10 min at 4 °C) in order to avoid unspecific antibody binding to HCMV-encoded Fc receptors. Flow cytometric analysis was performed using a BD LSR Fortessa (BD Biosciences) and FlowJo software (FlowJo Llc.) for data analysis.

### *In vitro* transcription and mRNA electroporation

The mRNAs encoding the HCMV-gB specific CAR and an gB/EpCAM fusion construct were obtained by *in vitro* transcription from a plasmid and a PCR product, respectively, as previously described^19^. Briefly, 1 µg of linearized plasmid or 50–200 ng of purified PCR product were used as template and transcribed using the mMessage mMachine T7 Ultra Kit (Invitrogen). According to the kit´s protocol, template DNA was mixed with T7 NTPs, T7 reaction buffer and T7 enzyme mix and filled up with nuclease-free water (all from Invitrogen) to a total reaction volume of 20 µl. After 1 hour of incubation at 37 °C, 1 µL of TurboDNAse (Invitrogen) was added and incubated further for 15 min at 37 °C to remove remaining template DNA. Afterwards, the poly-A-tailing reaction was set up using E-PAP buffer, 25 mM MnCl_2_ and ATP-solution (all from Invitrogen). This was added to the reaction tube and filled up with nuclease-free water to a total volume of 100 µL. After addition of the E-PAP enzyme (Invitrogen), the final incubation step was performed for 45 min at 37 °C. The resulting mRNAs were column purified with an adopted protocol using the RNeasy Kit (Qiagen). According to this protocol, RLT buffer from the kit and 1% beta-mercaptoethanol were added followed by addition of absolute ethanol. The mixture was loaded onto an RNeasy column and purification was performed according to the manufacturer’s protocol. Expression of gB-CAR in primary T cells was achieved by electroporation of 5 × 10^6^ T cells with 10 µg mRNA using the Square Wave protocol (500 V, 5 msec, 4 mm cuvettes) and Gene Pulser (Biorad). 293T cells were electroporated with varying amounts (0.1–10 µg) of EpCAM-gB mRNA using the same protocol with shorter pulse duration (500 V, 3 msec, 4 mm cuvettes).

### Degranulation assay

Degranulation of T cells was determined by extracellular staining of CD107a as previously described in more detail^19^ using an anti-CD107a antibody conjugated with PE (clone H4A3, BD biosciences). For this assay, 0.5 × 10^5^ target cells were blocked with human serum (10% final concentration) of an HCMV-seronegative individual for 10 minutes at 4 °C and 0.5 × 10^5^ effector cells were added (effector:target (E:T) ratio of 1:1) followed by addition of an anti-CD107a antibody. After 1 h of incubation at 37 °C, 5 µM Monensin was added to prevent internalization and the incubation was continued for 4 h. Afterwards, the cells were transferred to FACS staining tubes, washed and stained with antibodies against CD3, CD4 and CD8 as described above. Round-bottom 96-well plates were used for this assay to enhance cell-to-cell contact during the incubation period.

### Cytotoxicity assay

The effector cells in the cytotoxicity assay were CD8^pos^ T cells which were isolated from CD3/28 activated and expanded T cells by using the Dynabeads FlowComp Human CD8 Kit (Life Technologies). The non-radioactive europium release assay was used for analysis of the cytotoxic effector function of the T cells. Target cells were detached using 0.1 M EDTA pH 7.4 (37 °C, 10 min) and labelled with europium (Eu) according to the following procedure: Target cells were washed once in RPMI GlutaMAX and resuspended in 1 ml buffer containing 80 mM EuCl_3_ (Fluka, Buchs), 400 mM DTPA-Na_2_, 250 mg/ml dextransulfate, 50 mM Hepes (pH 7.4), 93 mM NaCL, 2 mM MgCl_2_ and 5 mM KCl (all from Merck). Cells were incubated in this buffer for 15 min at 4 °C on a rotating wheel. Afterwards, 20 ml of 100 mM CaCl_2_ were added and incubation was continued for 5 min followed by 3 washing steps in a buffer containing 50 mM Hepes (pH 7.4), 93 mM NaCl, 2 mM MgCl_2_, 5 mM KCl, 2 mM CaCl_2_ and 10 mM glucose. Cells were transferred to a tissue culture flask and incubated for 45 min at 37 °C while gentle shaking (80 rpm) to avoid attachment of the cells. Subsequently, cells were washed 2 times with phenol-free RPMI GlutaMAX (Life Technologies) and resuspended in the same medium supplemented with 10% FCS and 1% L-glutamine. After counting of the cells, 2500 labelled target cells were seeded in a volume of 100 µL in a 96-well round bottom plate and for the BiTE conditions, effector cells were added at an E:T ratio of 25:1. The gB-BiTE antibody construct (300 ng/ml) or T cells transfected with gB-CAR mRNA were added and cells were incubated for 4 h at 37 °C. The supernatant was analysed for Eu released from lysed target cells by addition of 200 µl Enhancement Solution (Perkin Elmer) to 25 µl co-culture supernatant using time-resolved fluorometry (TRF; Victor, Wallace). The specific lysis was calculated with the following formula:

$$\begin{array}{ccc}{\rm{specific}}\,{\rm{lysis}}\,[ \% ] & = & ({\rm{Eu}}\,{\rm{release}}\,{\rm{in}}\,{\rm{co}} \mbox{-} {\rm{culture}}-{\rm{spontaneous}}\,{\rm{Eu}}\,{\rm{release}})/\\  &  & ({\rm{maximum}}\,{\rm{Eu}}\,{\rm{release}}-{\rm{spontaneous}}\,{\rm{Eu}}\,{\rm{release}})\ast 100.\end{array}$$

### ELISA

For the generation of supernatants, effector and target cells were incubated at an E:T ratio of 2:1 in flat-bottom plates for 4 h at 37 °C. The supernatants were centrifuged (1600 rpm, 7 min) to remove remaining cells and frozen at −20 °C. For analysis of secreted cytokines, IFN-γ ELISA was performed using the Human IFN gamma ELISA Ready-SET-Go! (eBioscience) according to the manufacturer´s instructions. For detection of TNF, the Human TNF-α ELISA development kit (Mabtech) was used. The limit of detection for IFN-γ was 4 pg/ml and for TNF 13 pg/ml. For better illustration, the cytokine levels in most diagrams were normalized to the value achieved with the respective donor cells in a selected reference condition (same condition for all donors as stated in the figure legend, typically the one with the highest value). The reason for this was the strongly varying capacity of the different donor cells for producing cytokines. The normalized cytokine values were calculated using the following formula:

$$\begin{array}{c}{\rm{Normalized}}\,{\rm{value}}\,[ \% ]\,{\rm{in}}\,{\rm{condition}}\,{\rm{x}}={\rm{concentration}}\,[\mathrm{ng}/\mathrm{ml}]\,{\rm{in}}\,{\rm{condition}}\,{\rm{x}}/\\ \,\,{\rm{concentration}}\,[{\rm{ng}}/{\rm{ml}}]\,{\rm{in}}\,{\rm{the}}\,{\rm{reference}}\,{\rm{condition}}\,{\rm{of}}\,{\rm{the}}\,{\rm{respective}}\,{\rm{donor}}\ast 100.\end{array}$$

### Inhibition of HCMV replication in HFF by T cell supernatants

T cell supernatants were generated by co-culture (E:T 2:1, 4 hours, 37 °C) of CD3/CD28-activated T cells with non-infected or HCMV-infected HFF (AD169, MOI 5, day 4 p.i.) in the presence or absence of the BiTE antibody construct (300 ng/ml). Supernatants of those co-cultures were then added in serial dilutions (1:3, 1:10, 1:30) to HFF throughout and after infection (AD169, MOI 0.25, infectious supernatant removed after 4 hours by two times washing). After 4 days, the proportion of infected HFF was determined by flow cytometric quantification of the number of GFP^pos^ cells.

### Statistical analysis

Statistical significance was calculated using the paired two-tailed Student’s t test (***p < 0.001, **p < 0.01, *p < 0.05).

## Electronic supplementary material

Supplemental material

## Data Availability

Materials used in this study are available from the corresponding author upon reasonable request provided that (i) the provision of such material does not constitute an undue burden for the authors (e.g. due to the necessity to produce new material exclusively to meet the request) and (ii) the recipient signs a reasonable material transfer agreement.
