# Supplementary material for: A gB/CD3 bispecific BiTE antibody construct for targeting Human Cytomegalovirus-infected cells
Source: Sci Rep. 2018 Nov 28;8:17453. doi: 10.1038/s41598-018-36055-2 (PMC6261951; doi:10.1038/s41598-018-36055-2)
Supplement: Supplementary file 1 — Supplemental material [file 41598_2018_36055_MOESM1_ESM.pdf]

A gB/CD3 bispecific BiTE antibody construct for targeting Human Cytomegalovirus-infected cells

Charlotte U. Brey[1], Julia Proff[1], Natascha Teufert[2], Benjamin Salzer[1], Johannes Brozy[3], Markus Münz[3], Jochen Pendzialek[3], Armin Ensser[2], Wolfgang Holter[1,4] and Manfred Lehner[1,4*]

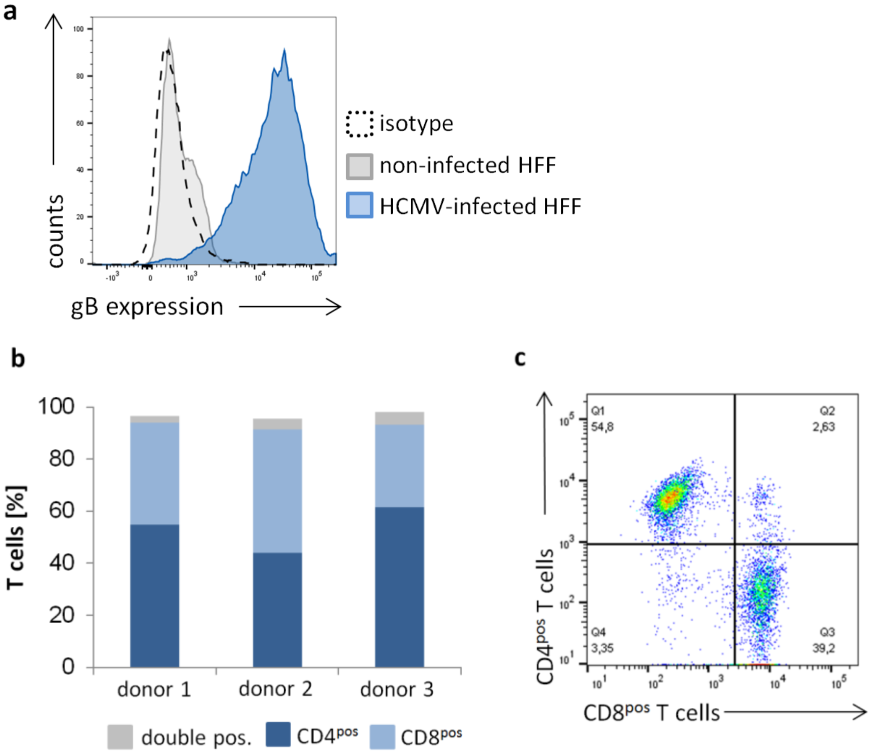

Supplemental Figure 1: (a) Expression of gB on the cell surface of HCMV-infected HFF (AD169, MOI 5, day 4 p.i.). Shown is one representative experiment (n=4). **(b)** Ratio of $CD3^{pos}/CD4^{pos}$ and $CD3^{pos}/CD8^{pos}$ T cells (gate was on viable cells) after expansion of negatively selected $CD3^{pos}$ cells of 3 different donors with Human T-Activator CD3/CD28 Dynabeads. **(c)** Representative dot plot of the flow cytometric analysis of the T cell phenotype (gate on $CD3^{pos}$ cells).

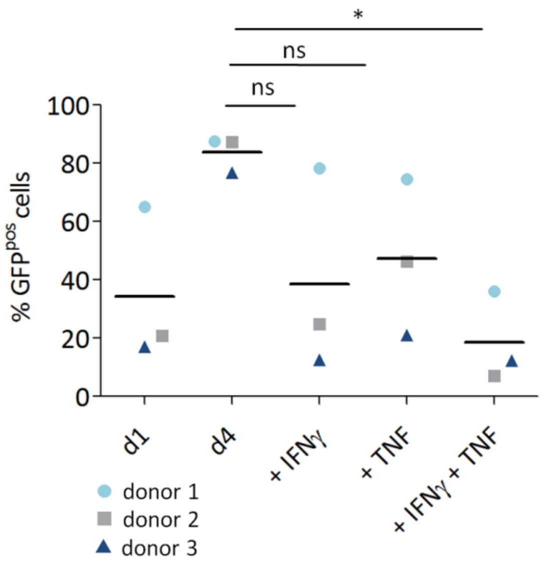

Supplemental Figure 2: Inhibition of HCMV replication in HFF by addition of IFN-γ (5 ng/ml) and/or TNF (5 ng/ml) during and after infection (AD169, MOI 0.3). The percentage of HCMV-infected HFF was determined by flow cytometric quantification of GFP^{pos} cells 4 days after infection.

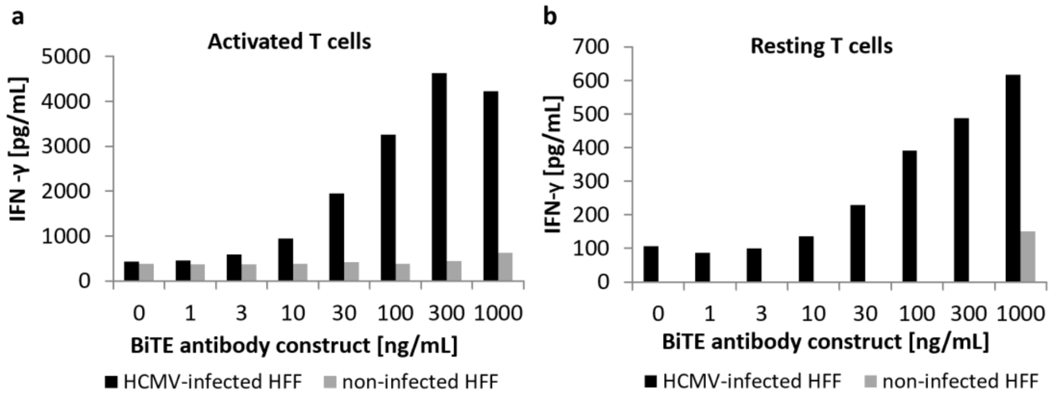

Supplemental Figure 3: The diagrams show the concentrations of secreted IFN-γ in the supernatants of co-cultures of either CD3/CD28-activated T cells **(a)** or resting T cells **(b)** with non-infected or HCMV-infected HFF (AD169, MOI 5, day 4 p.i.) and an increasing amount of gB-BiTE antibody construct, as indicated.

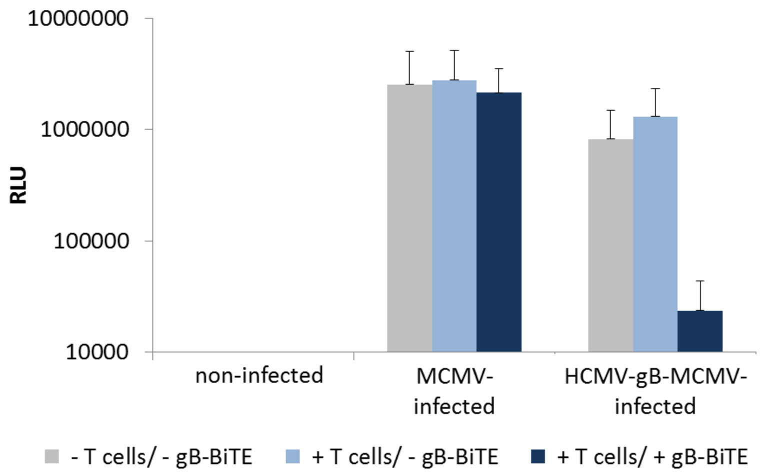

Supplemental Figure 4: BiTE antibody construct specific inhibition of a model MCMV recombinant virus *in vitro*. C57/BL6 murine embryonic fibroblasts were infected on d0 with the MCMV strain Smith m157-Luc[54] or a recombinant MCMV variant additionally expressing IE1-HCMV-gB and co-cultured with human CD3/CD28 expanded effector T cells in presence or absence of the gB specific BiTE antibody construct for 24 h. Virus spread in culture was determined by Luciferase-assay as described[54]; mean of 2 experiments (n=3).

REFERENCES

54. Hebeis BJ, Klenovsek K, Rohwer P, Ritter U, Schneider A, Mach M, et al. Activation of Virus-specific Memory B Cells in the Absence of T Cell Help. *J. Exp. Med.* **199,** 593-602 (2004).
